# Cell-Surface Integrins and CAR Are Both Essential for Adenovirus Type 5 Transduction of Canine Cells of Lymphocytic Origin

**DOI:** 10.1371/journal.pone.0169532

**Published:** 2017-01-09

**Authors:** Payal Agarwal, Elizabeth A. Gammon, Abdul Mohin Sajib, Maninder Sandey, Bruce F. Smith

**Affiliations:** 1 Scott Ritchey Research Center, College of Veterinary Medicine, Auburn University, Auburn, Alabama, United States of America; 2 Department of Pathobiology, College of Veterinary Medicine, Auburn University, Auburn, Alabama, United States of America; Swedish Neuroscience Institute, UNITED STATES

## Abstract

Adenoviruses are the most widely used vectors in cancer gene therapy. Adenoviruses vectors are well characterized and are easily manipulated. Adenovirus serotype 5 (Ad5) is the most commonly used human serotype. Ad5 internalization into host cells is a combined effect of binding of Ad5 fiber knob with the coxsackie virus and adenovirus receptor (CAR) and binding of RGD motifs in viral penton to cell surface integrins (αvβ3, αvβ5). Ad5’s wide range of host-cell transduction and lack of integration into the host genome have made it an excellent choice for cancer therapeutics. However, Ad5 has limited ability to transduce cells of hematopoietic origin. It has been previously reported that low or no expression of CAR is a potential obstacle to Ad5 infection in hematopoietic origin cells. In addition, we have previously reported that low levels of cell surface integrins (αvβ3, αvβ5) may inhibit Ad5 infection in canine lymphoma cell lines. In the current report we have examined the ability of an Ad5 vector to infect human (HEK293) and canine non-cancerous (NCF and PBMC), canine non-hematopoietic origin cancer (CMT28, CML7, and CML10), and canine hematopoietic origin cancer (DH82, 17–71, OSW, MPT-1, and BR) cells. In addition, we have quantified CAR, αvβ3 and αvβ5 integrin transcript expression in these cells by using quantitative reverse transcriptase PCR (q-RT-PCR). Low levels of integrins were present in MPT1, 17–71, OSW, and PBMC cells in comparison to CMT28, DH82, and BR cells. CAR mRNA levels were comparatively higher in MPT1, 17–71, OSW, and PBMC cells. This report confirms and expands the finding that low or absent expression of cell surface integrins may be the primary reason for the inability of Ad5-based vectors to transduce cells of lymphocytic origin and some myeloid cells but this is not true for all hematopoietic origin cells. For efficient use of Ad5-based therapeutic vectors in cancers of lymphocytic origin, it is important to address the defects in cell surface integrins.

## Introduction

Cancer is the second leading cause of human fatalities in United States [1]. Tumors of hematopoietic origin (Lymphoma, leukemia, mast cell tumor and myelodysplasia) comprise 9.4% of all cancer deaths in humans. In 2014, the estimated human fatality rate for diagnosed cases of lymphoma, leukemia, and myeloma was 25%, 46%, and 46% respectively [2]. In dogs, lymphomas represent 7–24% of all cancer diagnosed and 83% of all hematopoietic malignancies, while mast cell tumors are the most common (16–21%) cutaneous tumor [3]. High mortality rates in these tumors and rising case frequencies make new developments such as gene therapy in treating these cancers essential. Cancer gene therapy is the genetic approach to treat cancer cells by introducing tumor suppressor genes to replace inactivated endogenous genes of this type, downregulating oncogene expression, modifying tumor-specific immunity by introducing cell surface antigens to attract cytotoxic T cells, introducing prodrug convertase enzymes or using oncolytic viruses to kill tumor cells using vectors. Adenoviruses are an excellent choice of viral vectors for cancer therapeutics due to their high efficiency, broad range of host transduction, easy genome manipulation, non-integration into the host genome, potential payload capacity and their well characterized molecular biology. Adenovirus 5 (Ad5) is the most widely used viral vector in cancer gene therapies [4]. Ad5 infects cells by binding to the coxsackie and adenovirus receptor (CAR) followed by internalization mediated by binding of RGD motifs on the adenovirus penton base protein to transmembrane integrins (αvβ3, αvβ5) on the cell surface [5–7]. Following these interactions, the virus is internalized and transported to the nucleopore complex where the viral DNA is imported into the nucleus. Ad5 has no or minimal ability to transduce cells of hematopoietic origin, and thus cannot be used effectively for gene therapy in tumors of hematopoietic origin. Deficiency or absence of CAR receptors has been identified as a potential obstacle to the use of Ad5 for cancer gene therapy in many tumor types. Similarly, low levels of Ad5 infection in cells of hematopoietic origin in humans and mice have been linked to low CAR levels [8–11].

Since interaction and internalization of Ad5 with target cells is due the combined interaction with CAR and αvβ3 and αvβ5 integrins, we propose that a deficiency of cell membrane integrins (αvβ3, αvβ5) may be responsible for the lack of Ad5 infection in cells of hematopoietic origin. We have previously reported that low level of integrins on canine lymphoma cells are a potential obstacle to Ad5 infection by analyzing αvβ3 integrin expression levels in canine lymphoma cell lines and primary lymphoma cells [12]. In the current report, we have evaluated the infection pattern of an Ad5 based vector, Ad5GL, in a broader range of canine tumor cells including lymphomas, mast cells, melanomas, mammary adenocarcinomas, macrophages, normal canine fibroblasts and PBMCs and assessed the expression of both αvβ3 and αvβ5. Our data demonstrates the direct correlation of expression of integrins (αv, β3, and β5) at the molecular level with the ability of Ad5GL to infect the target cell.

## Materials and Methods

### Cell culture

Canine mammary tumor cell line CMT28, canine histiocytoma DH82, human embryonic kidney cells, HEK293, Canine melanoma cell lines CML7 and CML10, and primary normal canine fibroblast (NCF) cells were cultured in DMEM (Dulbeccos’s Modified Eagle’s Medium, Corning) with penicillin (100 IU/ml, Corning), streptomycin (100 ug/ml, Corning), amphotericin B (0.5ug/ml, Corning), non-essential amino acids (1X, Corning), and 10% FBS (fetal bovine serum, Sigma). Canine lymphoma cell lines 17–71 (gift of Dr. Steven Suter, North Carolina State University) and OSW (gift of Dr. William C. Kisseberth, The Ohio State University) and canine mast cell lines MPT1 (gift of Dr. Hiroshi Matsuda, Japan), and BR cells (gift of Dr. Cheryl London, Ohio State) were cultured in RPMI (Roswell Park Memorial Institute medium, Corning) with penicillin (100 IU/ml, Corning), streptomycin (100 ug/ml, Corning), amphotericin B (0.5ug/ml, Corning) and 10% FBS (Sigma). All cells were grown at 37°C (95% air; 5% CO2). All cells were confirmed by species specific PCR [13] to be canine cells except HEK293, which is human in origin, and by morphology and culture to be of the cell type indicated. MPT1 and BR were confirmed to be mast cells by staining with Toluidine Blue.

### Preparation of peripheral blood mononuclear cells (PBMC)

10 ml blood was drawn from the cephalic vein of a normal dog in accordance with the recommendations in the Guide for the Care and Use of Laboratory Animals of the National Institutes of Health. The procedure was part of a protocol was approved by the Auburn University Institutional Animal Care and Use Committee (PRN 2015–2688). Blood was collected in 10 ml 15% EDTA blood collection tubes (Tyco). Blood was centrifuged at 450g for 30 min at room temperature. After Centrifugation, the buffy coat layer was collected and transferred to 15 ml conical tubes (VWR). 2 ml of 1X PBS (Corning) was added to the buffy coat cells slowly. 5 ml of Histopaque 1077 (Sigma) was layered below the PBS/buffy coat cells mix. The layered suspension was centrifuged at 700g for 30 min at room temperature. The middle white cell layer was extracted post-centrifugation and collected in a 15ml conical tube with an equal amount of 1X PBS and centrifuged at 700g for 10 min at room temperature. The supernatant was removed and the white cell pellet was re-suspended in 5ml 1X PBS and centrifuged at 700g for 10 min at room temperature. After the centrifugation supernatant was removed, the PBMC (Peripheral Mononuclear Blood Cells) cell pellet was used for cellular RNA isolation using TRIzol reagent (Life Technologies) according to the manufacturer’s instructions.

### Virus infections

The Ad5GL virus vector, which encodes GFP (Green Fluorescence Protein) and Luciferase under the control of the cytomegalovirus (CMV) immediate early promotor (gift of Dr. David T. Curiel, St Louis, MO) was used for infections. Ad5G/L virus vector particle number was calculated by measuring optical density at 260 nm [14]. Virus infections were done at three different multiplicities of infection (MOI) (10, 100, and 1000 virus particles/cell). 1 X 10^5^ cells of CMT28, DH82, HEK293, CML7, CML10, and primary canine fibroblasts were plated in 24 well plates one day prior to virus infections. 1X10^5^ cells of 17–71, OSW, MPT1, and BR cell lines were plated the same day of infections. Cells were washed with 1X PBS (Phosphate Buffered Saline), and infected with 200ul of DMEM/RPMI (2% FBS), containing the virus. After one hour of infection, 400ul of DMEM/RPMI (10% FBS) was added to the cells. Cells were monitored at 24, 48, and 72 hours post infection for green fluorescence using an inverted fluorescent microscope (EVOS FL Cell Imaging System).

### Flow cytometry

All adherent cells were harvested and washed twice with 1X PBS. All the cells were re-suspended in flow wash buffer (1XPBS + 0.1% BSA; Bovine serum albumin) and analyzed for GFP expression by flow cytometry (Accuri C6).

### Preparation of RNA, primer design, and quantitative RT-PCR

Cell cultures were grown to 75–80% confluence and total cellular RNA was isolated using TRIzol reagent (Life Technologies) according to the manufacturer’s instructions. The concentration of RNA was determined by absorbance at 260 nm. Canine αv, β3, β5, CAR and beta-actin cDNA synthesis and amplification was performed by quantitative reverse transcriptase PCR (Q-RT-PCR) using specific primers (Table 1). All qPCR performed were conducted at 95°C for 3 minutes, and then 40 cycles of 95°C for 30 seconds and 57°C for 30 seconds. The specificity of the reaction was verified by melt curve analysis. Q-RT-PCR was performed using a Bio-Rad iCycler iQ Multicolor Real-Time PCR Detection System and assays were performed using SsoFast EvaGreen qPCR supermix (Biorad). mRNA expression was analyzed by the comparative ΔCt method. PCR products were purified using GeneJet gel extraction kit (Thermo) according to the manufacturer’s instructions and identity was confirmed by sequencing the amplicons (Eurofins MWG Operon).

**Table 1:** Primers used for Quantitative reverse-transcriptase PCR.

Gene	Primer (5’-3’)	Amplicon size (bp)	Genbank Accession No.*
**β-actin Forward**	ACGGGCAGGTCATCACTATT	220	NM_001195845.1
**β-actin Reverse**	ATCTCCTTCTGAATCCTGTCA		
**CAR Forward**	GCCAGCTACGCCTGAATGTT	189	XM_005638767
**CAR Reverse**	TCTTAGGAGGCGGCACATCT		
**Beta-5 Forward**	TCAGATGGACTATCCGTCCCTT	131	XM_846152.3
**Beta-5 Reverse**	TTGTCCCAGGTATCAGGGCT		
**Beta-3 Forward**	CGGCGTCGGAGTGTCCAA	192	NM_001003162.1
**Beta-3 Reverse**	ACCTCACTGATGGGGAACTCA		
**Alpha V Forward**	GGCGATGGCGTAGATGACTT	197	XM_845896.3
**Alpha V Reverse**	GGCGCTCCGATGAACACAT		

Primers were designed using NCBI Primer blast. Primers are based on canine sequences

*published in Genbank.

### Statistical analysis

Multiple linear regression analysis was performed at the 95% confidence level to analyze the functional relationship between infectivity of human adenovirus serotype 5G/L as determined by the percentage of cancer cells expressing GFP by flow cytometry compared to the relative expression levels of αV, β3, β5 and CAR by various canine cancer cell lines.​

## Results

### Ad5GL virus infections

**Fig 1:**
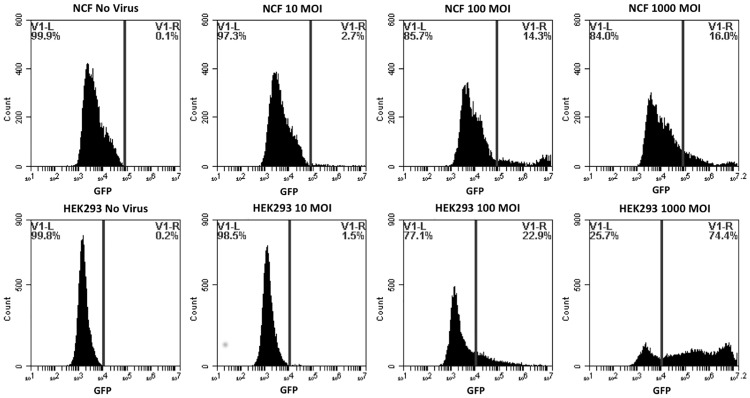
GFP Expression by non-cancerous normal canine fibroblasts (NCF) and HEK293 cells. Cells were transduced by adenovirus Ad5G/L at three different multiplicity of infection (MOI 10, 100, and 1000 virus particles per cell). GFP expression in cells was analyzed by flow cytometry 48 hours after Ad5G/L infection.

**Fig 2:**
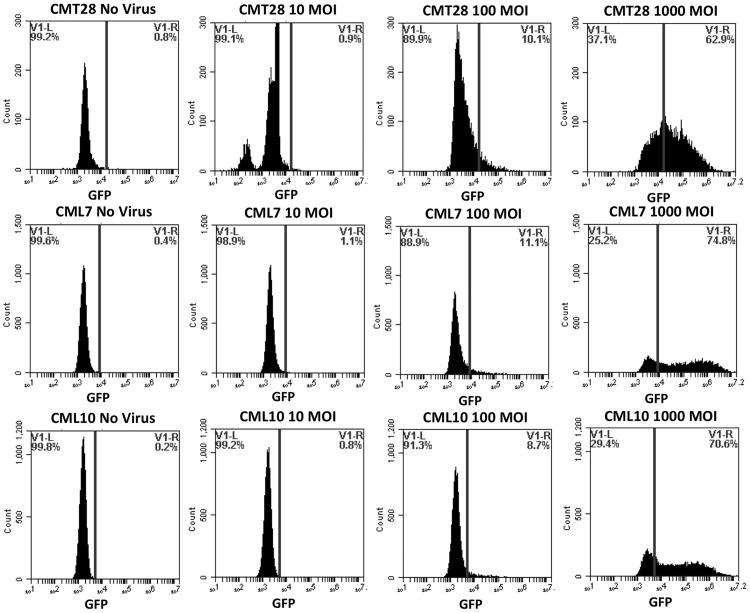
GFP Expression by canine non-hematopoietic origin cancer cells; Canine Mammary Tumor (CMT28), and Canine Melanoma (CML7 and CML10). Cells were transduced by adenovirus Ad5G/L at three different multiplicity of infection (MOI 10, 100, and 1000 virus particles per cell). GFP expression in cells was analyzed by flow cytometry 48 hours after Ad5G/L infection.

**Fig 3:**
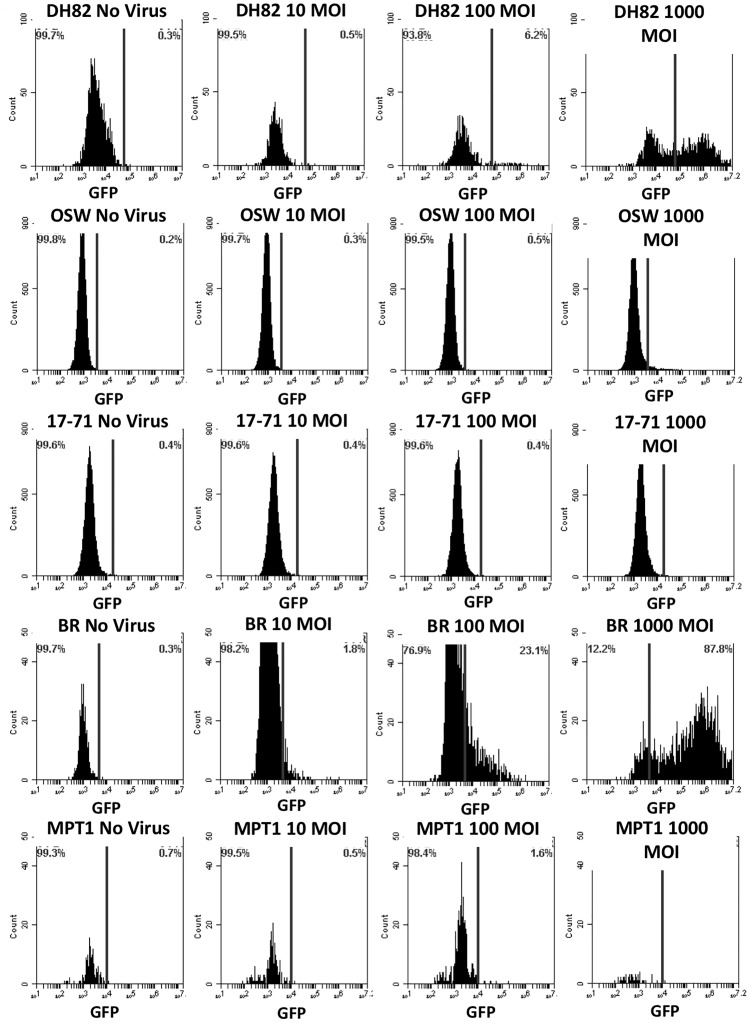
GFP Expression by canine hematopoietic origin cancer cells; Histiocytoma (DH82), Lymphoma (1771 and OSW) and Mast Cell Tumor (MPT1 and BR) cells at three different MOIs. Cells were transduced by adenovirus Ad5G/L at three different multiplicity of infection (MOI 10, 100, and 1000 virus particles per cell). GFP expression in cells was analyzed by flow cytometry 48 hours after Ad5G/L infection.

Primary canine fibroblasts, adherent non-hematopoietic cancer cells (CMT28, CML7, CML10), adherent hematopoietic cancer cells (DH82), non-adherent hematopoietic cancer cells (MPT1, BR, 17–71, OSW), and positive control HEK293 were infected with a reporter adenovirus construct, Ad5GL, which has wild-type Ad5 tropism and expresses green fluorescent protein from the CMV immediate early promoter. Cells were examined by fluorescent microscopy (data not shown) and flow cytometry (Figs 1–3) to determine the number of cells expressing the GFP reporter gene post Ad5G/L transduction. HEK293, NCF, cancerous non-hematopoietic origin CMT28, CML7, and CML10 cells, hematopoietic DH82 cells, and mast cell tumor line BR all expressed green fluorescence 48 hours post Ad5GL infection at MOIs of 100 and 1000 virus particles/cell (Figs 1–3). This was evident by the increased percentage of Ad5G/L transduced cells showing levels of fluorescence over the background of auto fluorescence in comparison to non-transduced cells. However, non-adherent cancerous cell lines of hematopoietic origin, MPT1, 17–71, and OSW, had very low (4.9% in OSW cells) or no GFP expression, even at the highest virus concentration (1000 MOI) (Fig 3). The percentage of cells expressing GFP in Ad5G/L infected cells is presented in Table 2. When the percentage of cells expressing GFP in comparison to non-infected cells was calculated the rate of infection was proportional to the virus particle MOI, except for MPT1, OSW, and 17–71 cells. Virus infection rates in susceptible cell lines varied, ranging from 16% in NCF at MOI 1000 to 87.8% in BR at MOI 1000. The flow cytometry data showed that Ad5G/L readily infected non-cancerous cells and cancerous cell of non-hematopoietic and hematopoietic origin while most cancerous cell of hematopoietic origin were either poorly infected, or not infected at all. The mast cell line BR and the histiocyte line DH82 were the exception among cells of hematopoietic origin and were readily infected by Ad5GL (87.8% and 55% respectively at 1000 MOI).

**Table 2:** Percent of Cells infected by Ad5G/L.

	0 MOI (Percentage Population)	10 MOI (Percentage Population)	100 MOI (Percentage Population)	1000 MOI (Percentage Population)
**NCF**	**0.1**	**2.7**	**14.3**	**16**
**HEK293**	**0.2**	**1.5**	**22.9**	**74.4**
**CMT28**	**0.8**	**2.7**	**10.1**	**62.9**
**CML7**	**0.4**	**1.1**	**11.1**	**74.8**
**CML10**	**0.2**	**0.8**	**8.7**	**70.6**
**DH82**	**0.3**	**0.5**	**6.2**	**55**
**BR**	**0.3**	**1.8**	**23.1**	**87.7**
**MPT1**	**0.7**	**0.5**	**1.6**	**1.8**
**OSW**	**0.2**	**0.3**	**0.5**	**4.9**
**17–71**	**0.4**	**0.4**	**0.4**	**0.6**

Quantitative GFP expression by non-cancerous (NCF, HEK293, and PBMC), non-hematopoietic origin cancer (DH82, CMT28, CML7, and CML10), and hematopoietic origin cancer (17–71, OSW, MPT-1, and BR) cells using BD Accuri^™^ C6 Cytometer software.

### Relative quantification of alpha V, beta3, beta5, and CAR mRNA

**Fig 4:**
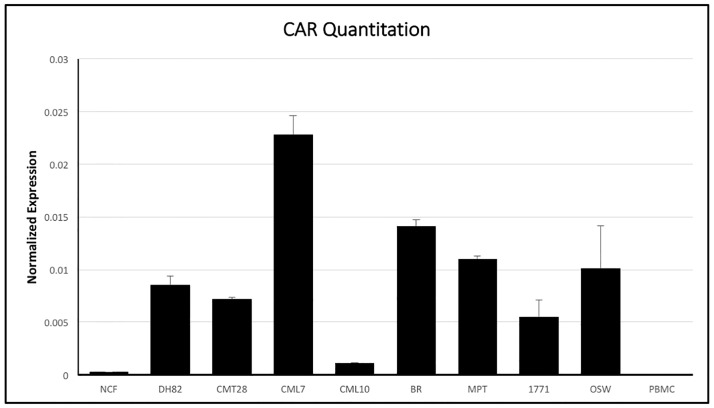
Quantitative RT-PCR analysis of coxsackievirus and adenovirus receptor (CAR) mRNA expression in canine non-cancerous (NCF and PBMC), non-hematopoietic origin cancer (CMT28, CML7, and CML10), and hematopoietic origin cancer (DH82, 17–71, OSW, MPT-1, and BR) cells using specific primers. CAR mRNA expression levels were normalized to levels of control β-actin mRNA expression and quantified using the ΔCt method.

**Fig 5:**
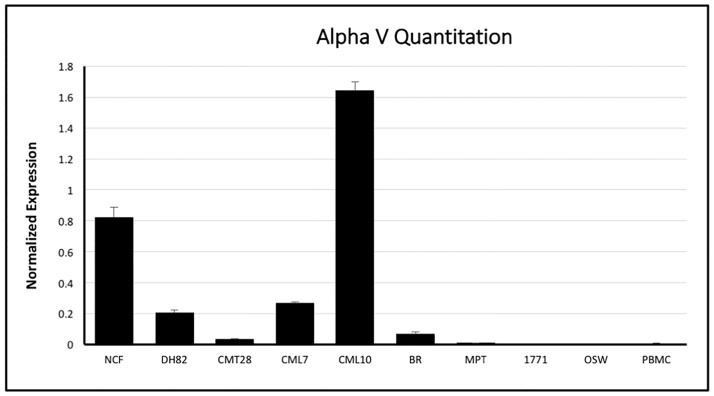
Quantitative RT-PCR analysis of alpha V membrane integrin mRNA in canine non-cancerous (NCF and PBMC), non-hematopoietic origin cancer (CMT28, CML7, and CML10), and hematopoietic origin cancer (DH82, 17–71, OSW, MPT-1, and BR) cells using specific primers. Alpha V mRNA expression levels were normalized to levels of control β-acting mRNA expression and quantified using the ΔCt method.

**Fig 6:**
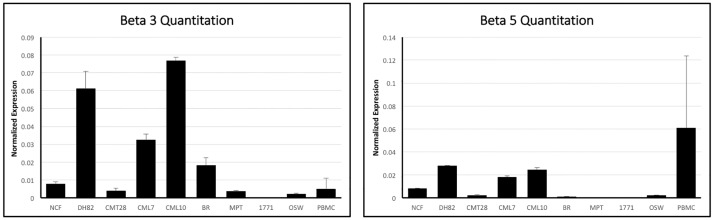
Quantitative RT-PCR analysis of beta-3 and beta-5 membrane integrin mRNA in canine non-cancerous (NCF and PBMC), non-hematopoietic origin cancer (CMT28, CML7, and CML10), and hematopoietic origin cancer (DH82, 17–71, OSW, MPT-1, and BR) cells using specific primers. beta-3 and beta-5 mRNA expression levels were normalized to levels of control β-actin mRNA expression and quantified using the ΔCt method.

Ad5 infection is dependent on the availability of CAR and cell surface integrins αvβ3 and αvβ5 on target cells. Initial attempts to analyze expression of these receptors on the cell surface, using antibodies derived against human integrins and CAR, were unsuccessful, as these antibodies did not reliably or quantitatively cross react with canine cells. In order to better understand the mechanism for Ad5 resistance, the expression of CAR, αv, β3 and β5 mRNA was determined using quantitative reverse transcriptase PCR using the comparative ΔCt method by normalizing mRNA expression of CAR, αv, β3 and β5 to the house-keeping gene β-actin’s mRNA expression in all cells. CAR mRNA was expressed in almost all of the cells tested, however the level of expression varied (Fig 4). The hematopoietic origin lines DH82, BR, MPT1, 17–71 and OSW all expressed CAR at moderate to high levels, comparable to the non-hematopoietic derived CMT28 and CML7 cell lines. NCF and CML10 showed relatively low mRNA expression in comparison to the other cells tested and PBMCs showed very low or no CAR mRNA expression. NCF, DH82, CML10, and CML7 expressed αv integrin mRNA at high or moderate levels, while CMT28 and BR expressed this integrin at moderate to low levels and MPT1, 17–71, OSW and PBMCs expressed αv integrin at very low levels (Fig 5). β3 expression patterns were similar to β5 mRNA expression with DH82, CM10 and CML7 expressing these integrins at high levels, NCF and CMT28 expressing both at intermediate levels and MPT1, 17–71 and OSW expressing both at very low levels (Fig 6). BR cells expressed β3 at moderate levels and β5 at low levels while PBMCs expressed β3 at moderate to low levels and β5 at very high levels.

In order to determine if the infection by Ad5 could be statistically correlated with the expression of αV, β3, β5 and CAR, multiple linear regression analysis was performed. When analyzed independently, the percentage of infected cells at MOI 1000 was dependent on the relative expression of β3 (p value: 0.0196) and CAR (p value: 0.0127). However, no statistical correlation could be identified between infection of cells and the expression of αV (p value: 0.548) and β5 (p value: 0.148). When a model including interaction between CAR and β3 was used, there was also strong positive correlation (p value: 0.0327) between the relative expression of CAR, β3 and the ability of Ad5 to infect cancer cell lines. In addition, β5 shows strong correlation in this model (p value: 0.0107) while αV has a p value: 0.196).

## Discussion

Conventional cancer therapies are often non-specific, resulting in the killing of non-cancerous cells along with cancer cells. The consequences of this are all too clear in the form of severe side effects in patients undergoing therapy. Therefore, alternative treatments where the cancer cells are specifically targeted are an important and pressing need.

Replication-deficient and conditionally-replicative adenoviruses are a valuable cancer gene therapy tool. More than 50 adenoviruses have been identified and have been classified according to their genetic relatedness, their tissue tropism and the disease syndromes they cause. Adenoviruses have a core of double stranded DNA chain with 35,000–37,000 base pairs covered by a capsid made of hexon, penton base, and fiber proteins. These viruses transduce a wide variety of dividing and non-dividing cells naturally with their tropism depending on the adenovirus fiber and the matching cellular targets. Adenoviruses are easy to produce with high functional titers and have a large packaging size. These characteristics make adenoviruses the most commonly used vector in clinical trials to treat both cancer and non-cancerous diseases [12, 15]. Adenoviruses are therapeutically important for many types of gene therapies and are not limited to cancer gene therapy. Due to these characteristics, adenoviruses are an appropriate choice for many gene therapy approaches.

Adenoviruses are an important tool to specifically target cancer cells, either as a gene delivery agent, or as a means to lyse cancer cells. Adenoviruses may be either replication defective (deleting E1 region) or conditionally replicative (E1A expression controlled by tissue-specific promoter). Adenovirus 5 is the most extensively used adenovirus and has been employed to treat cancers like prostate, lung, head and neck, skin, central nervous system, ovarian, breast, kidney, and many other cancer types [16–23]. Ad5 has also been used to treat cardiovascular diseases such as ischemia and coronary artery diseases and infectious diseases [24, 25]. However, in some cases the effect is reduced due to inefficient transduction owing to strong innate or pre-existing immune responses against the virus or inefficient targeting of adenovirus due to lack of cellular receptors. The latter is especially true for diseases of hematopoietic origin, such as lymphoma, leukemia, mast cell tumor, etc. [9, 11].

Infection of target cells by wild type Ad5 requires a two-step process whereby Ad5 transduces the target cell by attaching to CAR and internalizes by interacting with cell surface αvβ3 and αvβ5 integrins [5–7]. The inability of Ad5 to infect hematopoietic cells has been attributed to poor CAR receptor expression on these cells.

Our initial experiments confirmed that Ad5G/L efficiently infected normal human (HEK293) and canine (NCF) cells as well as a variety of cancer cells of non-hematopoietic and hematopoietic origin, including canine mammary tumor (CMT28), melanoma (CML7 and CML10) histiocytoma (DH82), and mast cells (BR). We also showed that Ad5G/L was unable to transduce other cancer cells of hematopoietic origin including canine lymphoma (17–71 and OSW) and mast cell tumor (MPT1) cells.

Based on these findings, CAR, αvβ3 and αvβ5 expression on these cells was evaluated. Neither CAR nor the associated integrins could be assessed at the protein level for expression in our cells and cell lines due to the lack of antibodies with demonstrable cross reactivity to canine cells expressing these proteins. As a consequence, CAR and integrin expression was examined using quantitative RT-PCR. These findings confirmed that cells that were transduced by Ad5 such as NCF, CMT28, DH82, CML7, CML10 and BR cells express CAR at varying but detectable, levels. These cells also express all three integrins (αv, β3, β5) at detectable levels. Although poorly infected by Ad5, MPT1, 17–71 and OSW cells, express CAR at relatively high or moderate levels. However, these cells express all three integrins at low or non-detectable levels, indicating that the block to infection is at the level of internalization. Interestingly, unlike the tumors of hematopoietic origin, PBMCs did not express CAR at all. PBMCs did express both β3 and β5 integrin but did not express αv integrin. This lack of αv integrin would lead to the failure to express either the αvβ3 or αvβ5 complexes on the cell surface. Thus, infection by Ad5 in PBMCs appears to be blocked at two steps, both viral attachment to CAR and viral entry. In an attempt to validate these findings by a second approach, the GEO database was searched for quantitative expression data for the cells and cell lines in this study. Affymetrix Canine Genome 2.0 array data for CAR, alpha-v, beta-3 and beta-5 in 17–71, OSW, DH82 and CML10-C2 cells (a clonal line related to but not exactly the same as the CML10 cells used) was identified [26]. When normalized with beta-actin, this data showed no significant differences in the expression of these genes. Given that qRT-PCR is considered the gold standard by which Affymetrix data must be verified, the significance of the Affymetrix data is unclear.

Our result shows that CAR expression does not vary drastically in tumor cells of hematopoietic origin in comparison to non-hematopoietic cells. However, the level of integrin mRNAs are low or absent in hematopoietic derived tumor cells that are unable to transduce by Ad5G/L in comparison to non-hematopoietic cells and other hematopoietic cells (MPT1) that can be transduced by Ad5G/L. Statistical analysis confirmed that expression of CAR, β3 and β5 were strongly correlated with Ad5G/L transduction. Thus, the inability of Ad5 to infect lymphocytic and some hematopoietic derived cancer cells can be attributed to the lack of integrin expression rather than CAR expression on the target cells. We also propose that blockade of Ad5 infection is not a property of all cells of hematopoietic origin, but is limited to lymphocytic cells and mast cells. One cell line, BR, stands in contradiction to our findings of the other mast cell line, MPT1. This cell line has been confirmed to be a mast cell tumor, however, it is significantly more de-differentiated than the other mast cell line examined, MPT-1. This dedifferentiation may have led to the expression of CAR, αvβ3 and αvβ5 allowing infection by Ad5.

In the search to optimized therapeutic approaches for gene therapy, it is important for a vector to transduce target cells efficiently while retaining target specificity. Thus, it was important to ascertain the mechanism resulting in the inability of Ad5 to infect cells of lymphocytic origin and mast cells. Our results suggest that the presence of both CAR and αvβ3 and αvβ5 integrins must both be evaluated and in the case of retargeted vectors, the functions of both must be included in some manner. In this way, both viral adhesion to the cell and viral entry into the cell can be accomplished.
